# Surface microstructures developed on polished quartz crystals embedded in wet quartz sand compacted under hydrothermal conditions

**DOI:** 10.1038/s41598-021-94376-1

**Published:** 2021-07-21

**Authors:** Peter M. Schutjens, Christopher J. Spiers, André Rik Niemeijer

**Affiliations:** 1 Shell Global Solutions International B.V., Rijswijk, The Netherlands; 2 HPT Laboratory, Faculty of Geosciences, Utrecht University, Utrecht, The Netherlands

**Keywords:** Geochemistry, Geology, Mineralogy, Petrology, Sedimentology

## Abstract

Intergranular pressure solution plays a key role as a deformation mechanism during diagenesis and in fault sealing and healing. Here, we present microstructural observations following experiments conducted on quartz aggregates under conditions known to favor pressure solution. We conducted two long term experiments in which a quartz crystal with polished faces of known crystallographic orientation was embedded in a matrix of randomly oriented quartz sand grains. For about two months an effective axial stress of 15 MPa was applied in one experiment, and an effective confining pressure of 28 MPa in the second. Loading occurred at 350 °C in the presence of a silica-saturated aqueous solution. In the first experiment, quartz sand grains in contact with polished quartz prism ($\overline10{1 }0$) faces became ubiquitously truncated against these faces, without indenting or pitting them. By contrast, numerous sand-grain-shaped pits formed in polished pyramidal ($17\overline{6 }3$) and ($\overline{4 }134$) crystal faces in the second experiment. In addition, four-leaved and (in some cases) three-leafed clover-shaped zones of precipitation formed on these prism faces, in a consistent orientation and pattern around individual pits. The microstructures observed in both experiments were interpreted as evidence for the operation of intergranular pressure solution. The dependence of the observed indentation/truncation microstructures on crystal face orientation can be explained by crystallographic control of stress-induced quartz dissolution kinetics, in line with previously published experimental and petrographic data, or possibly by an effect of contact orientation on the stress-induced driving force for pressure solution. This should be investigated in future experiments, providing data and microstructures which enable further mechanism-based analysis of deformation by pressure solution and the effect of crystallographic control on its kinetics in quartz-rich sands and sandstones.

## Introduction

Intergranular pressure solution (IPS) is a deformation mechanism whereby interfacial and diffusive mass transfer occurs through an aqueous phase present within grain boundaries and pores, facilitating compaction creep of porous rocks and deviatoric creep of dense rock materials (see^1^ for a review). The process is driven by an effective stress acting on fluid-infiltrated granular aggregates^2^. Stress concentrations at grain contacts induce gradients in chemical potential within the solid and thus create local dis-equilibrium between the solid and the solute in solution. Dissolution occurs preferentially where the chemical potential of stressed solid is higher than that of the solute in solution; precipitation occurs where the chemical potential of the solute in solution is higher than that of the solid. In a system where there is no long range advective transport of solute in or out of the system by flowing pore fluid, the gradient in solute concentration leads to material diffusion from the high-stress sites of dissolution (the grain contacts) to the low-stress sites of precipitation, through the fluid-infiltrated grain-to-grain contacts and the fluid in the pores. Dissolution and diffusion of material from highly stressed grain contacts to contacts supporting lower stress, or to free pore walls, produces a change in grain shape and a relative displacement of neighboring grains centers, which leads to creep deformation and/or compaction, depending on stress state and porosity. The process is variously termed intergranular pressure solution (IPS), pressure solution, pressure solution creep, or fluid-assisted diffusion creep.

It is generally accepted that characteristic mass removal microstructures, like grain-to-grain indentations, contact truncations and sutured grain contacts in rocks, are the result of IPS (see^1^ for a review). Because of its effectiveness in reducing intergranular porosity, IPS is considered one of the main factors controlling the geologic evolution of porosity and permeability, and hence capacity and productivity of hydrocarbon and geothermal reservoir sandstones^3,4^ as well as the capacity and injectivity of potential CO_2_ or H_2_ storage reservoirs^5^. Alongside reducing porosity, IPS tends to increase the average surface area of grain contacts, which enhances the load-bearing capacity (failure strength) and reduces (poro-elastic) compressibility. The extent (i.e. the "intensity") of IPS in sandstones appears to depend on thermal maturity/history, grain size (e.g.^6^) and crystallographic orientation with respect to dissolution contact surface (e.g.^7,8^), as well as on a number of other factors which are still poorly understood. These include pore fluid composition, concentrations of clays and micas in grain contacts and pores^9,10^ and the presence of grain contact cement phases. Equally important is the role of IPS in controlling the compaction, cementation and hence healing and sealing behavior of fault gouges formed in faults cutting both clastic sedimentary sequences and granitic basement (e.g.^11,12^). IPS has also been shown to be a key mechanism controlling the frictional behavior of fault gouges and in particular the unstable shearing behavior involved in the nucleation of both natural and induced earthquakes^13^.

The above interest in IPS processes has provided an incentive for the development of numerous microphysically-based constitutive equations addressing compaction and deformation by IPS creep (e.g.^14–17^) as well as its effects on fault gouge compaction and shear^13^. Two broad classes of grain scale mechanisms have been proposed. The first is the so-called marginal dissolution process^18^, envisaged to involve progressive undercutting of grain contacts (Supplementary Fig. 1, top). The second is grain boundary diffusion (e.g.^17^), involving dissolution and diffusion of material through a very thin (adsorbed) fluid film (Supplementary Fig. 1, middle) or an intergranular fluid-filled island-channel network (Supplementary Fig. 1, bottom), with the additional possibility of microcracking at grain contacts providing an additional diffusion path (see e.g.^3,9^).

Indentation/truncation and overgrowth microstructures have been observed in wet quartz sands compacted at effective pressures of several tens to hundreds of MPa and temperatures up to 300 – 500 °C, and these features have been interpreted as evidence for the operation of IPS^4,11,19^. Similar microstructures have been observed in (simulated) quartz and quartz-phyllosilicate fault gouges deformed in laboratory experiments settings^20^. However, because of the post-mortem nature of the observed microstructures (i.e. representing the end-product of deformation) and insufficient information on initial and final grain boundary structure, the intergranular dissolution mechanism, i.e. grain boundary diffusion in a thin film or island-channel structure, vs. marginal dissolution and/or contact crushing, remains difficult to identify and may involve several or all of the above. The two experiments reported in this paper were directed at shedding light on the mechanisms operating at stressed grain contacts during IPS in quartz. To accomplish this, we investigated *changes* in the morphology of contacts between quartz grains and polished, single crystal quartz "plates" and “blocks” (here termed inclusions) embedded within fluid-saturated (“wet”) quartz sand samples compacted at hydrothermal conditions. So, only the plate and the general grain size/shape of the sand were known, not the initial contact geometry at grain-plate contact.

As explained below, in the two experiments, we used different quartz sand materials, different apparatus setups and different effective stresses. This makes comparison and unambiguous interpretation of the results difficult, but the experiments nonetheless produced interesting and rather surprising microstructures which (we hope) will stimulate further work where experimental variables will be systematically varied.

## Experimental method

### Sample preparation and compaction method—experiment QC13

#### Single crystal quartz inclusions

Quartz cylinders with a diameter of 1 cm and a length of 1.5–2 cm were cored from inclusion-free Brazilian quartz crystals in directions parallel to the crystallographic r-axis and m-axis (Supplementary Fig. 2). Cylindrical quartz discs with a thickness of 1.5–2 mm thickness were prepared by cutting perpendicular to the long axis of the cylinder. Half-cylinders and quarter-cylinders were prepared by cutting parallel to the long axis of the tube. The saw-cut surfaces were first manually ground using abrasive paper and then automatically polished for about 24 h on progressively finer abrasive paper (Emery 4/0) in the presence of a water-based SiC paste. This procedure resulted in plate- and block-like inclusions with a high-quality, virtually scratch-free, surface finish (Supplementary Fig. 3a). The dimensions of the quartz crystal inclusions used in the present tests and the crystallographic orientations of the polished surfaces are listed in Table 1 and the experimental conditions in Table 2.

**Table 1:** Geometry of experiment and dimensions and orientation of polished faces on single crystals of quartz.

Experiment	Geometry	Dimensions of polished faces (mm)^1^	Crystallographic orientation^2^
QC 13	Cylinder at bottom of sand sample	Φ = 10, h = 1.6–1.8	($10\overline{1 }0$)
QC 14	Quarter of a cylinder Face A Face B	h = 9.0, w = 2.0 h = 9.0, w = 3.0	($17 \overline{6 }3$) λ = 203, θ = 64 ($\overline{4}314$) λ = 315, θ = 48

^1^φ = diameter, h = height, w = width.

^2^λ = azimuth, θ = dip, λ = 0º: crystallographic [1010] direction, θ = 0º: crystallographic [0001] direction.

**Table 2:** Experimental conditions.

Experiment	QC13	QC14
Temperature (°C)	350	350
Maximum applied confining pressure, Pc (MPa)	27.5	45.0
Maximum vapour pressure, Pf-max (MPa)	15.5	17.0
Maximum effective pressure, Pe-max (MPa) ^1^	15.0	28.0
Experiment duration (days)	62	61
Initial sample volume (mm^3^)	5937.6	124.5
Volume of polished crystal(s) plus sand (mm^3^)	3207.5	82.8
Initial pore volume (mm^3^) ^2^	2730.1	41.7
Initial porosity (%) ^3^	46.0	33.5
Initial pore fluid composition	1000 ppm SiO_2_ (aq)	d.d. H_2_O SiO_2_ (aq)
Liquid volume added at 20 °C (mm^3^)	All pores filled with brine	10.5

^1^Calculated from vapour pressure data^21^, assuming liquid plus vapour present in the capsule. Pressure of included air is assumed negligible.

^2^Inner volume of the capsule minus the volume of quartz.

^3^A lower porosity was achieved for QC14 by gently agitating the gold capsules during and for 5 min after filling with sand.

#### Quartz sand

Quartz sand obtained from the Miocene "Bolderiaan" formation (Maasmechelen, Belgium) was used as indenter material. The sand was sieved into a grain size fraction of 125–150 µm, ultrasonically vibrated to remove adhering fines, washed with distilled water and then etched in a HF solution (0.5–1 N, duration ~ 20 min) to remove possible surface damage ). After the HF treatment, the sand was successively rinsed with NaOH solution of decreasing strength (0.5 N, 0.01 N and 0.001 N**),** washed with de-ionized water, and then dried for 3 days at 45 °C. Most quartz grains were characterized by crystallographically controlled surface patterns of cusp-shaped asperities (0.5–1 µm) and triangular, pyramidal and sickle-shaped etch pits (0.1–0.5, µm, Supplementary Fig. 3b). Most grains were rounded or at least displayed rounded edges, probably due to the etching by HF preferentially dissolving sharp edges.

#### Sample assembly and compaction procedure

A single cylindrical quartz plate was included at the base of sand sample QC13, formed by depositing the crystal and sand into a 1-D piston-cylinder (oedometer) type compaction vessel. The polished top surface ($10\overline{1}0$) was oriented orthogonal to the cylindrical axis of the sample and vessel, so that the applied (total) axial stress was transmitted to this quartz face via the overlying quartz grains (the matrix, see Supplementary Fig. 3c). Axial compaction was conducted using a piston-cylinder (oedometer) type compaction vessel under wet conditions (see definition below) at 350 °C, for 62 days, under effective axial stresses stepped in three increments of 5 to 15 MPa. In such an oedometer test, the effective radial stress is not known, yet it can be safely assumed that some effective radial stress (i.e. support pressure) is exerted on the grains above and at the quartz crystal interface. The details of the vessel and of the experimental procedure are described by Schutjens^22^. The pore fluids were prepared by dissolving analytical grade sodium-metasilicate salt (Na_2_Si0_3_·5H_2_0) at 20 °C in double-distilled water to obtain a solution of 1000 ppm SiO_2_, which is the equilibrium concentration with respect to quartz at the desired testing temperature and fluid pressure (calculated using data compiled by^23^). This was done to minimize dissolution of the quartz sand by any mechanism other than stress-related solution transfer effects.

### Sample preparation and compaction method—experiment QC14

#### Single crystal quartz inclusion

The preparation procedure of the quartz crystal faces in test QC14 was identical to that in QC13. In QC14, however, quarter-cylinders were used instead of cylindrical discs (see Supplementary Fig. 2). The polished quartz faces (referred to here as polished facets) were parallel to crystal planes ($17\overline{6}3$) and ($\overline{4}314$).

#### Quartz sand

In experiment QC14, quartz grains from the weakly consolidated St. Peter sandstone from the Ordovician (provenance USA) were used as indenters. A sieved fraction of 125–150 µm was ultrasonically cleaned in tap water and dried for 2–3 days at 45 °C. Most grains were well-rounded. The following surface morphologies were distinguished. Firstly, the largest part of the surface (60–70%) was found to be characterized by a mosaic-pattern of tabular, relatively flat surfaces (width: 2–5 µm) separated by randomly oriented micro-channels of about a micron in diameter (Supplemental Fig. 4a). The remainder of the surface of the quartz grains showed a crystallographically controlled pattern of cusp-shaped and serrated ridges with a relief of 1–3 µm (Supplemental Fig. 4b).

#### Sample assembly and compaction procedure

Sample QC14 was compacted hydrostatically in a closed system containing the quartz inclusion with two polished facets, quartz sand and water in liquid and vapour phase (Supplemental Fig. 4c). To achieve this, the quartz single crystal inclusion was placed in a gold capsule (outer diameter 6 mm, wall thickness 0.2 mm) and the quartz sand was funneled around the crystal. The capsule was agitated during and for some 5 min after filling with sand, in order to obtain a denser packing of the grains. The capsule was closed with a gold lid and welded along the contact between capsule and lid, leaving a small opening. After weighing the capsule (accuracy: ± 0.1 mg) the volume of air inside the capsule was calculated. Knowing the air volume and assuming a sand porosity under stress of about 25%, a calculation was made of the volume of water required to maintain water vapour present next to liquid water at the testing temperature of 350 °C. The presence of both liquid and vapour water in a closed hydrothermal system allows estimation of the pore fluid pressure (= the vapour pressure of water at the testing temperature), which is then more or less independent of the amount of quartz sand compaction. Based on the standard tables of the liquid–vapour equilibrium of water at elevated temperature^21^, 10.5 ml of water was added to the sample using a syringe inserted through the opening at the top of the capsule. The opening was closed by pinching the lid flap against the inner wall of the capsule, the capsule was weighed to determine the amount of water added and then weld-sealed. Evaporation of the water due to welding was minimized by mounting the capsule on top of a copper heat-sink immersed **i**n liquid nitrogen. The welded capsule was tested for leakage by immersion in oil at a temperature of 120 °C. The dimensions were measured with a caliper. The filled capsule was placed in an externally heated Tuttle pressure vessel and compacted hydrostatically using Argon gas as a pressure medium. The confining pressure (P_c_) was applied by manually opening and closing the link valve between the pressure vessel and an independently pressurized Argon reservoir—and was measured with a 50 MPa full scale pressure transducer. Every 6 to 12 h, P_c_ was increased in steps of about 3 MPa until a final value of 45 MPa was reached. After each P_c_-increment, the temperature of the vessel was increased by 20 to 30 °C. The pore fluid pressure at a given temperature was taken as the vapour pressure at that temperature, assuming liquid water to be present in the capsule at every stage during the experiment. Based on the liquid–vapour data of water at elevated temperature^21^, the confining pressure increments were chosen such that the corresponding increase in confining pressure was somewhat greater than the increase in pore fluid pressure. In this way, the effective confining pressure was gradually increased from zero MPa at 20 °C to about 28 MPa at 350 °C (i.e. with a confining pressure of 45 MPa and the vapour (pore) pressure of about 17 MPa). For a period of 61 days, the capsule was maintained at a constant P_c_ of 45 MPa, a constant (inferred) pore pressure of 17 MPa and a constant temperature of 350 °C. After termination of the experiment, the quartz single crystals were taken from the vessel (QC13) and capsule (QCI4) with a rubber-coated pair of tweezers, washed in de-ionized water, dried and prepared for microstructural analysis.

## Experimental results

### Results of experiment QC13

#### Aggregate microstructure

**Figure 1:**
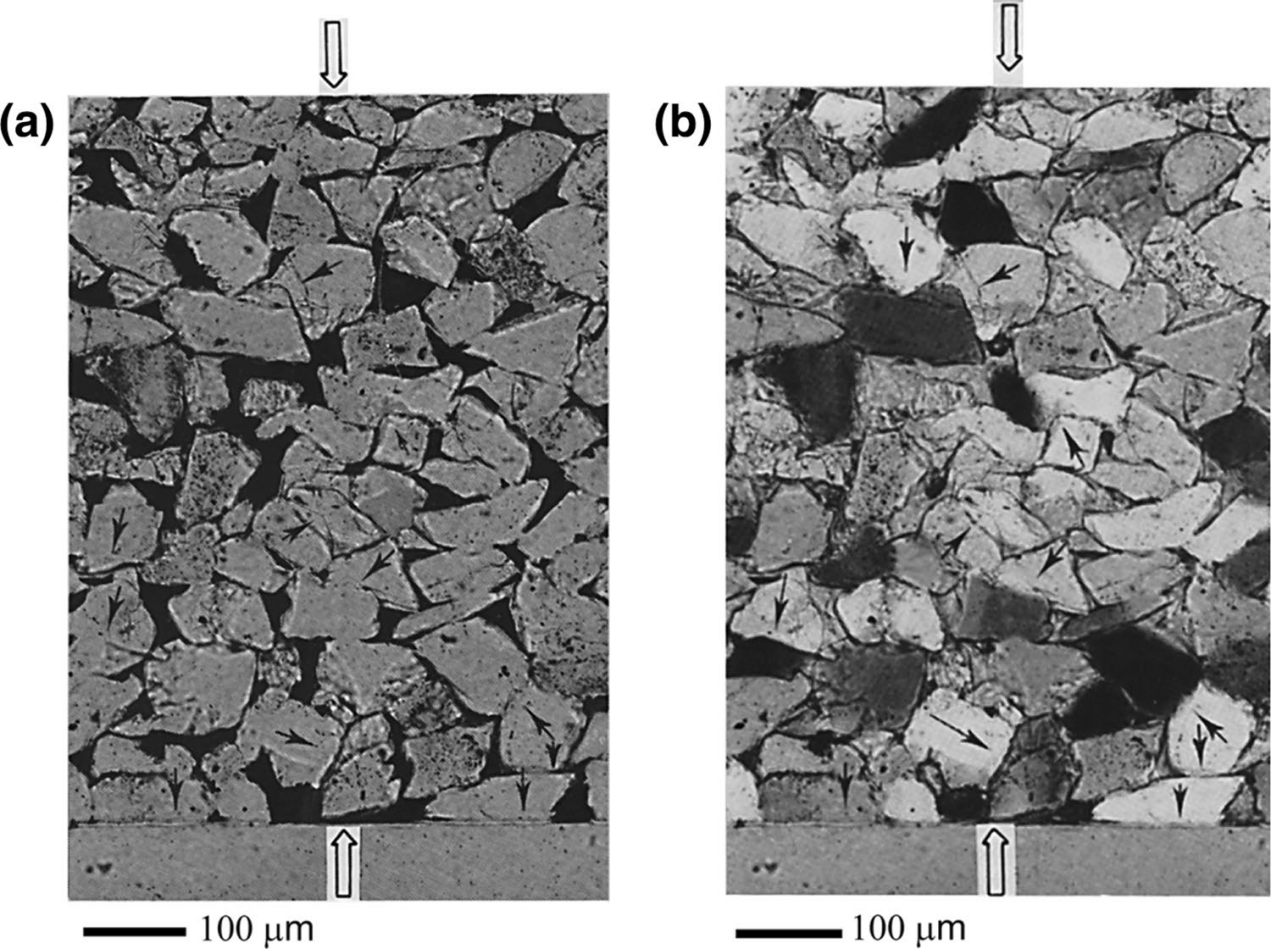
Overview photomicrographs of quartz sand compacted wet at 350 °C in test QC13. (**a**) Plane polarized light, with pores colored black. (**b**) Crossed polars. Both photomicrographs show the same part of a thin section cut parallel to the cylindrical axis of the sample (i.e. parallel to the direction of the applied effective stress, indicated by the open arrows). Notice the grain contact truncation and grain indentation microstructures (thin arrows). Just visible at the bottom is the polished cylindrical quartz crystal. Note conspicuous grain contact truncation of quartz grains from the matrix against this crystal.

**Figure 2:**
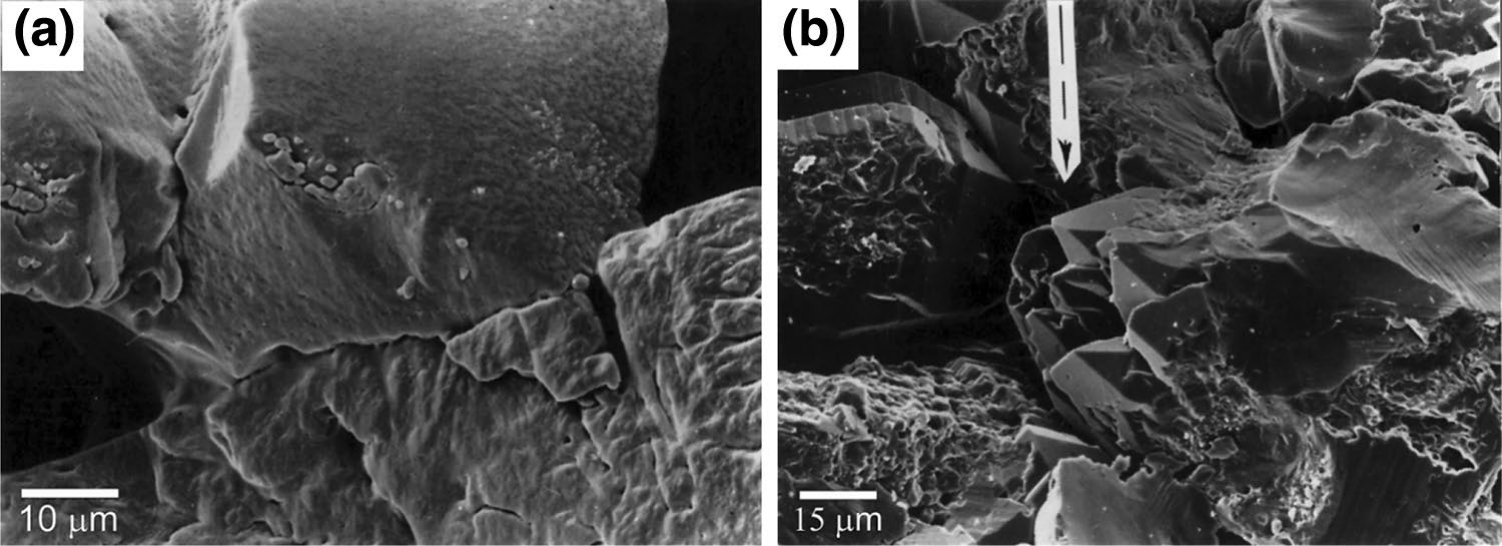
(**a**) Characteristic grain contact truncation microstructure in wet-compacted sample QC13 (T = 350 °C). Note that only one of the two grains in contact appears to have undergone dissolution. This feature is observed at nearly all the truncated/indented grains. Note also the absence of marginal dissolution features. (**b**) Euhedral quartz crystals in the pores of the wet-compacted sample QC13.

**Figure 3:**
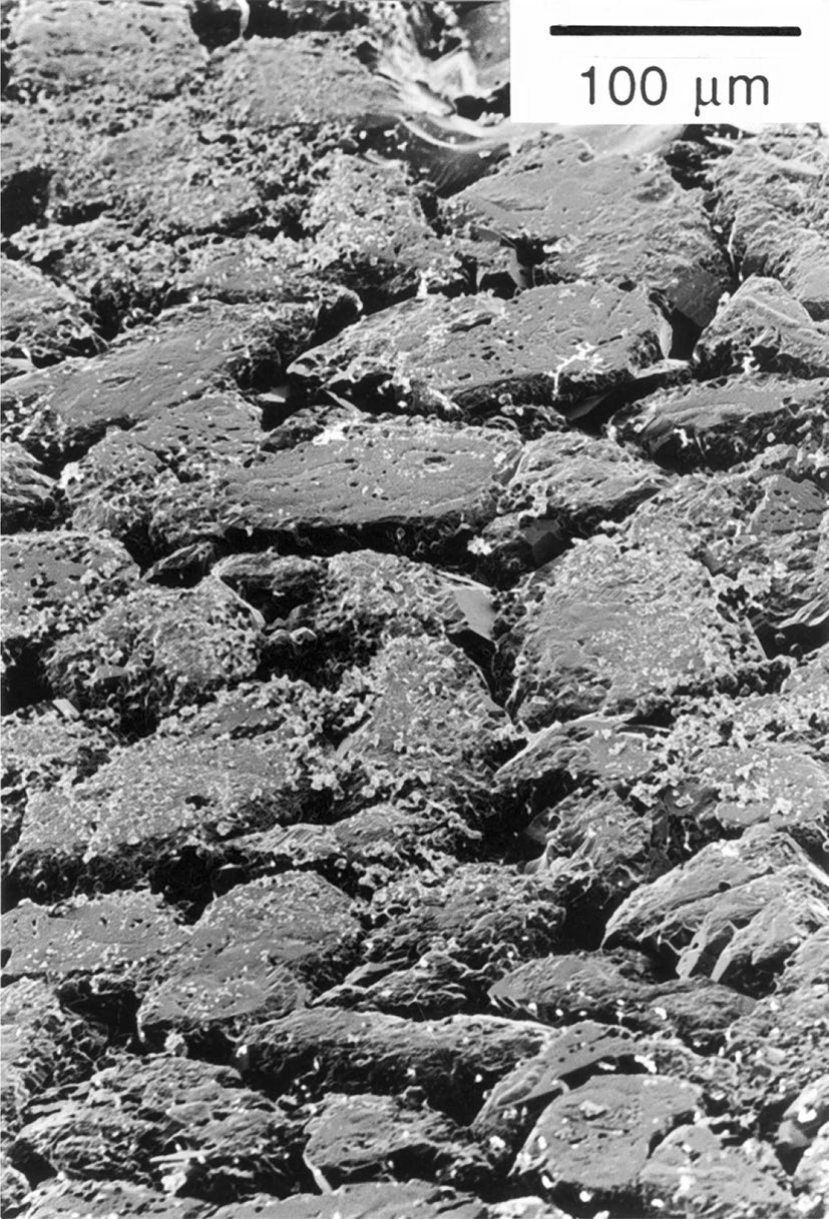
SEM micrograph showing top surface of the truncated quartz crystals after removal of the polished quartz plate. Note flat top surface of the grains.

Figure 1a,b shows optical micrographs of the compacted sample of experiment QC13 (the oedometer test). The polished quartz single crystal is visible at the bottom of the photographs. Abundant truncated and concavo-convex grain-to-grain contacts were observed in the matrix of Bolderiaan quartz grains located *above* the polished quartz crystal (Fig. 2a, i.e. at contacts between individual quartz grains), as well as clusters of euhedral quartz crystals in the pores (Fig. 2b). Microstructural evidence was found for truncation of matrix quartz grains against the face of the polished quartz crystal located at the base of the sample (Fig. 3) but no pitting of quartz grains into the crystal was observed with the optical microscope.

#### Surface microstructure of ($10\overline{1 }0$) face

**Figure 4:**
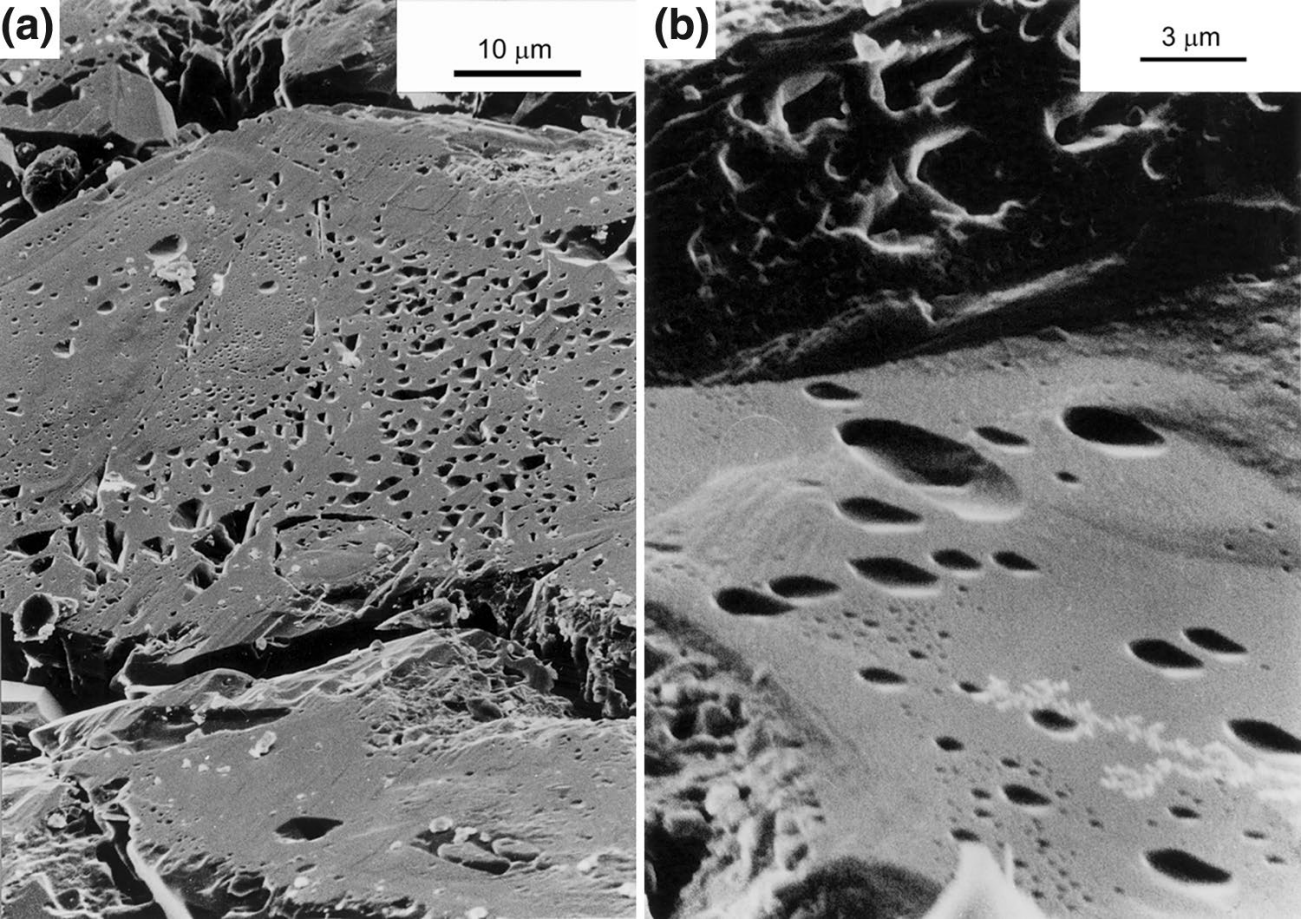
(**a**,**b**) SEM micrographs of truncation interfaces of the grains which were in contact with the quartz plate. Note the inclusion morphologies and the flat intervening regions between the apparently isolated inclusions.

**Figure 5:**
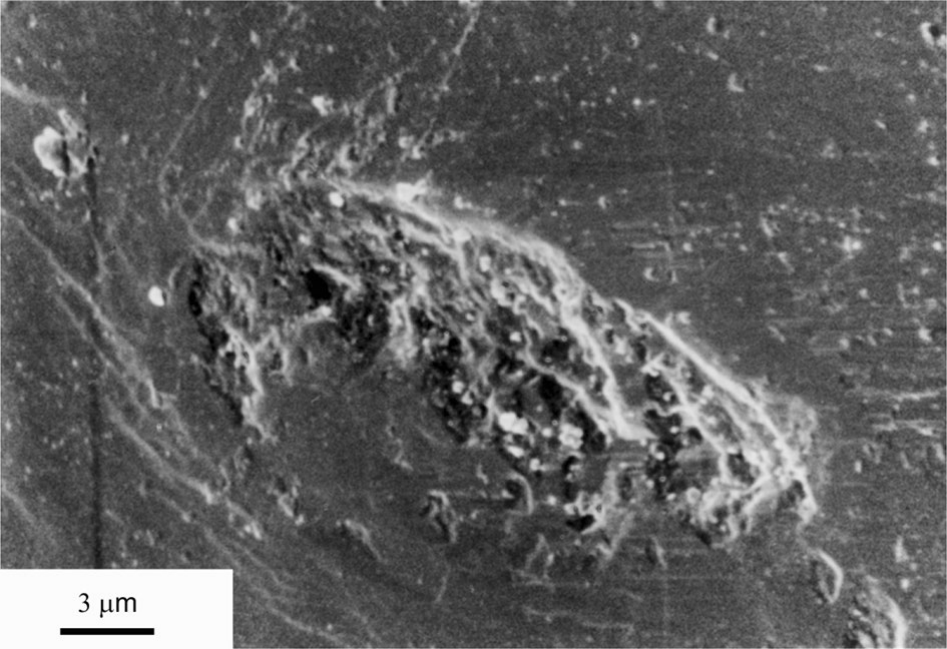
Indentation in the polished crystal face used in test QC13. Only a few of these indentations were observed; most matrix crystals which were in contact with the polished quartz crystal face were truncated without indenting the polished crystal face. Note fine grained debris in and around the pit (inferred to have formed during compaction).

Figure 4a,b show SEM micrographs of the contact surface morphology of quartz grains which were pressed against the polished quartz crystal face. Note the conspicuous flat top surface (estimated roughness < 500 nm), confirming the impression gathered from optical microscopy of widespread truncation of the quartz grains against the polished ($10\overline{1}0$) face. The flat top faces display a wide variety of inclusions, varying in shape from triangular and inverse-pyramidal (Fig. 4a) to sub-rounded (Fig. 4b), and ranging in size from several hundred nanometers to about 3–4 µm. The intervening regions between the inclusions appear to be flat within SEM resolution, which implies that the surface relief (if any) in these regions is smaller than about 10 nm. SEM observations confirmed that pits into the polished crystal surface are virtually absent. The few pits that were seen with the SEM are oval-shaped and have a long dimension in the range 3 to 10 µm (i.e. relatively small compared to the size of the pits developed on face ($\overline{4}314$) in experiment QC14, see below). The boundary between pits and the polished surface is indistinct, and numerous submicron-size particles are visible at and around these pits (Fig. 5, white-colored in view). These must have formed during experimental compaction or during cooling, since the starting surface of the quartz crystals was free of adhering particles. No overgrowths were seen on the quartz plate, but limited overgrowths features did develop on quartz grains in the sample, see Fig. 2b.

### Results of experiment QC14

**Figure 6:**
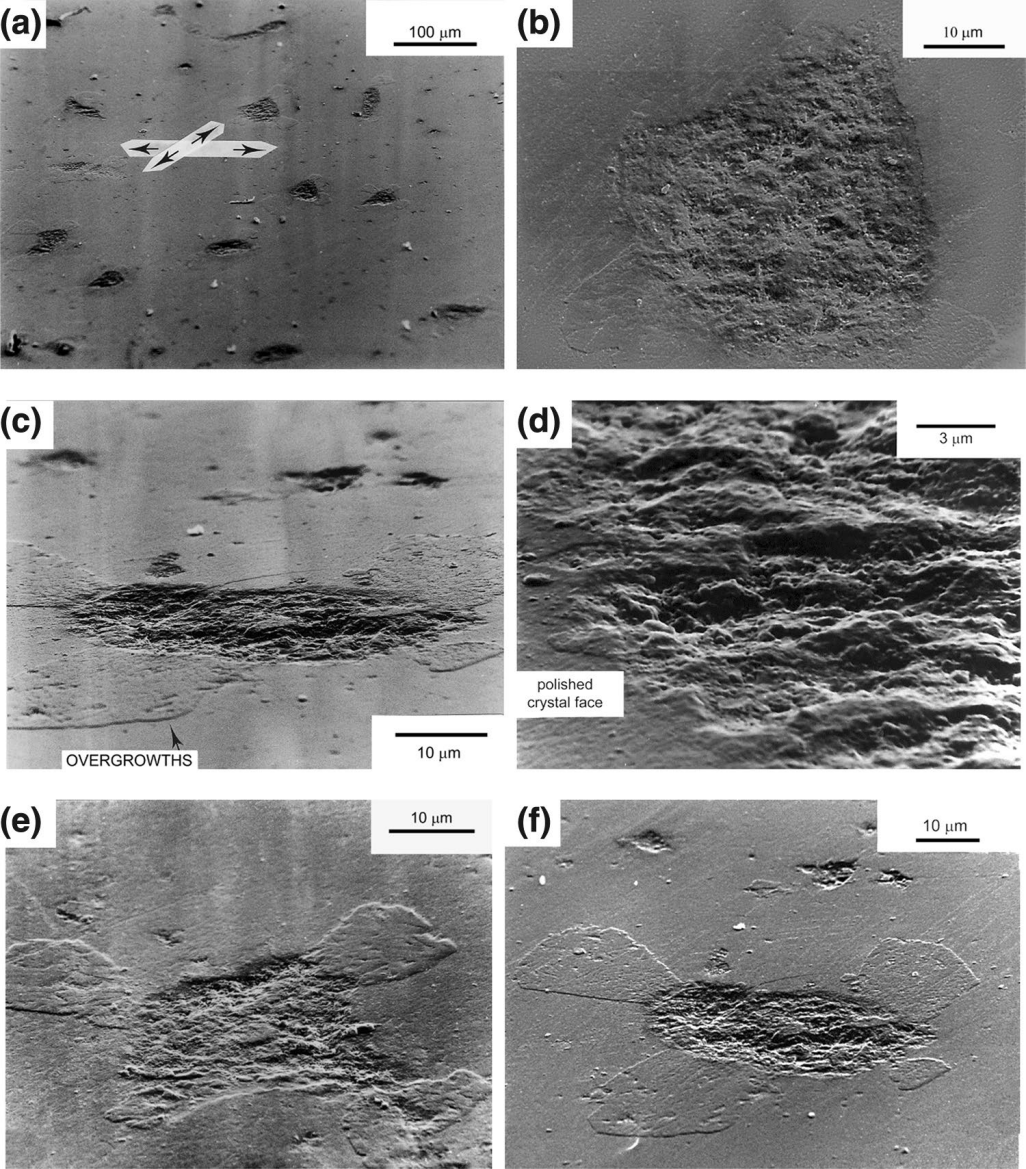
Micrographs of “pits” surrounded by clover-shaped precipitation microstructures, developed on the polished quartz crystal in experiment QC14. (**a**) SEM micrograph of pits on crystal face ($\overline{4}314$), birds-eye view. (**b**) Micrograph of pit taken perpendicular to a pit surface. crystal face ($\overline{4}314$). (**c**) Birds-eye view of pit on face ($\overline{4}314$). (**d**) Micrograph showing the typical rough surface structure of the pit and the sharp transition between the "rough" pit and the '"smooth" polished crystal face. (**e**,**f**) Typical "4-leaf clover" geometry of overgrowth developed around pits. Note the different size of the “clovers”. Also note the shape preferred orientation of the overgrowth features in Fig. 6a (indicated by arrows).

The extracted gold capsule was crumpled around the sample and clear imprints of the quartz grains had developed on the inner wall of the capsule. SEM micrographs of the polished quartz faces included in this test are shown in Fig. 6. Abundant sub-elliptical and occasionally sub-circular pits formed on the polished surface with orientation ($\overline{4}314$). Their long dimension lays in the range 30 to 60 microns and is apparently randomly oriented. In most pits, the depth of the pits below the polished surface was found to gradually increase towards the center of the pit to estimated values of 3 to 10 µm ("bowl" shaped pits). In other pits, the depth below the surface is more or less constant over the entire surface area. Figure 6b shows a typical pit morphology viewed perpendicular to the polished quartz-crystal face. Views at low angles to both types of pits reveal a relatively homogeneous surface morphology characterized by a micro-relief of blunted knobs, ridges and furrows, occasionally cross-cut by zones showing cuspate ("sharp") surface features (Fig. 6c). From SEM observations, we estimate that the relief ranges from several hundred nanometers up to a few micrometers, with an estimated average of 1 µm. Note the distinct transition from the "smooth" polished surface to the "rough" surface of the pit (Fig. 6d).

The indenter grains corresponding to the pits were not recovered, and so the actual amount of mass removal associated with formation of a given pit is unknown. On the same polished crystal face ($\overline{4}314$) many pits were seen to be bordered by tabular elevations with a typical lobate shape (Fig. 6c, see arrow at base). Because the starting surface was perfectly flat, material is inferred to have precipitated during hydrothermal compaction and will therefore be referred to here as "overgrowth". Though hard to determine precisely, the overgrowth features have an elevation of about 1 µm above the polished surface (this is our coarse estimate from inspection of the photographs), cover surface areas in the range of 200–500 µm^2^ and appear to be in crystallographic continuity with the substrate crystal (since no euhedral overgrowths were seen). Pits show in general four overgrowths, occasionally three, but rarely more than four. One of the overgrowths is often considerably smaller than the others (as in Fig. 6c,e,f). The longest dimension of the individual overgrowths (measured perpendicular to the rim of the pit) is nearly always oriented at an angle of 90° to that of the neighboring overgrowth, giving rise to a characteristic "4-leaf clover" geometry, with the pit in the center. The orientation of the clover-leaf overgrowth geometry on the polished face is constant with respect to an external reference frame (indicated by arrows in Fig. 6a). The size of the pits seems to be in qualitative agreement with the size of the bordering overgrowths: the bigger the pit, the bigger the overgrowths around it. No attempt was made for quantitative petrography to gather data to study and map the size and distribution patterns of the indentation-overgrowth microstructures.

The pits developed on polished face ($17\overline{6}3$) are about twice as small (average diameter 20–30 µm) and less deep (5 µm) than those developed on the ($\overline{4}{314}$) face, as descbed above (see Supplemental Fig. 2). The surface relief of the pits on ($17\overline{6}3$) is also less pronounced than on ($\overline{4}{314}$) but is of similar morphology. Limited evidence was also found for "clover-leaf' overgrowths around these pits, again with the longest dimension of the individual overgrowths (measured perpendicular to the rim of the pit) being oriented at an angle of 90° to that of the neighboring lobate overgrowth.

## Interpretation

### Deformation mechanism

The following observations are of interest to identify the mechanisms active during hydrothermal compaction in tests QC13 and QC14:

1. The pits in the polished quartz surfaces form at the locations where the grains from the matrix are in contact with the quartz crystal plates, and are thus not due to dissolution variation caused by e.g. along-surface variation in dislocation density due to polishing or impurities in the quartz crystal.
2. The shape of most pits is rounded to sub-rounded (see Fig. 6b) with their depth gradually increasing towards its pit-center. The pit-surface rugosity is not smooth and probably reflects the morphology of the indenting quartz grains. This indicates that material has been removed by dissolution rather than pushed aside after progressive brittle deformation. In that case, probably a more irregular pit surface with sharp ledges and fractured parts would have formed.
3. Because no evidence was found for pit development by fracturing and because plastic deformation of quartz can be ruled out at these relatively low PT conditions^24^, it is inferred that the pits seen in QC13 and QC14 were formed by stress and/or strain-induced solution transfer (i.e. IPS) from the contact points between polished crystal facets and quartz grains. We think that the truncation microstructures observed in the axial compaction test QC13 are also indicative of IPS.
4. QC13 and QC14 were conducted at similar temperature (350 °C), similar applied effective pressure (a few tens of MPa) and similar duration (two months). Despite the difference stress application (axial loading in oedometer vs hydrostatic loading) and different surface morphology of the quartz sand, IPS was observed in *both* tests. Mechanism-based grain-scale models for pressure solution predict IPS to occur at these conditions^4,15^.
5. The morphology of the pits seen in test QC14 suggests that they formed by uniform indentation of the St. Peter quartz grains into the polished faces, and not by some mechanism of free-face dissolution around the small ("undercut") points of contact. In the latter mechanism, a tabular "neck"-shaped contact would be expected, surrounded by regions with a flat or crystallographically-controlled surface morphology (as proposed by^18^. This was not seen in our samples.
6. The precipitation on the polished quartz faces occurs only where the pits are (see Fig. 6e,f), providing additional evidence that the pits formed by dissolution.
7. The "clover-leaf” geometrical configuration around the pits and their uniform orientation on the quartz crystal face (see Fig. 6b) suggests control by the internal structure of the quartz crystal on their formation. Quartz has a trigonal symmetry and is mechanically anisotropic, with the a-axis being ~ 50% more compressible than c-axis^25^. Differences in the dissolution rates of different crystal faces of quartz have been implied from both field work (e.g.^8^), experimental studies (e.g.^26^) and theoretical analysis (e.g.^27^). The clover-leaf overgrowth pattern with orthogonal directions could also be caused by crystallographically controlled strain energy variation across the quartz-crystal face, i.e. defining regions of relatively low and high stress (“stress shadows”) or regions of relatively high/low dislocation density that act as locations for preferential precipitation (“step propagation from dislocation defect”^28,29^).
8. The observation of truncation against the (1010) quartz crystal face in test QC13 and of indentation in the (17 $\overline{6 }$ 3) and ($\overline{4}314$) faces in test QC14 points to control of crystallographic orientation on IPS.
9. The pits that developed in test QC14 on polished face (1763) are about twice as small (average diameter 20–30 µm) and less deep (5 µm) than those developed on the ($\overline{4}314$) face, as described above. This also suggests control by the quartz crystal lattice on the rate of IPS.
10. The sub-rounded to crystallographically controlled shape of the inclusions at the truncated quartz grains seen in test QC13 (Fig. 4a,b) resemble grain boundary structures from natural sandstones where IPS occurred (^3^, their Fig. 4) and suggest the operation of surface-energy-driven solution and precipitation inside the grain contact interface. Inspection of the truncated contacts in test QC13 (Fig. 4b) suggests that the surface roughness is below 500 nm. This seems to favor the thin-film hypothesis although some “dynamic” island-channel microstructure inside the quartz-quartz contact cannot be ruled out.

Summarizing, it is inferred that the indentations in tests QC13 and QC14 and truncations in test QC13 formed by a process of grain boundary diffusional mass transfer, with the effective grain boundary width governed by the surface structure of the indenting quartz grains, with 1 µm as upper limit and less than 500 nm at the "flat" regions like the ones shown in Fig. 4a.

### Comparison to experiments of Renton and Gratier

In test QC14, evidence for IPS at the quartz-crystal facets was provided by the presence of overgrowths close to the indentations. Stress trajectories on an indenter-loaded quartz surface likely take the shape of clover-leafs, at least for near-spherical indenters like the Bolderiaan and St. Peter quartz grains, with modification due to the anisotropic elastic properties of the quartz crystal. Assuming that the clover leafs are indeed elevations and not depressions and assuming that the concentration of dissolved silica is radially symmetric, the areas covered by the clover leafs must be areas of low strain energy density, presumably controlled by the anisotropic elastic strain field around the indenter.

Similar indentation features were observed by Renton et al.^19^ in hydrostatic compaction experiments using polished faces of quartz crystals surrounded by small (inert) zircon grains in the presence of a fluid phase. In their experiments, elliptical to sub-rounded pits were produced on the polished quartz faces, with the extent of pit formation increasing with pH and salinity of the pore fluid. The surface morphology of their pits was also rough, but instead of a random distribution of protuberances, ridges and furrows (as in the present experiments) surface relief was characterized by an annular pattern composed of alternating ridges and valleys and by radial solution grooves. Renton and coworkers also observed overgrowth features at the rims of the pits,—but mention no particular shape of these overgrowths. The fact that the observed overgrowths formed at the rims of the indentations led Renton and coworkers to conclude that the solute did not move far from the site of dissolution. Our observations, including a qualitative correlation between pit and overgrowth volume, support this conclusion.

Steel indenter experiments in quartz crystal surfaces by Gratier and co-workers demonstrated the occurrence of stress-induced axially symmetric cracks below the surface of the indenter and extending out into the crystal face^16^. In their experiments, the axially symmetric cracks acted as fluid channels leading to free face dissolution. We did not observe any evidence for such micro-cracking at the quartz surfaces, but microcracks may have been present *inside* the quartz crystal (this could be investigated with thin section analysis, which we did not do) or microcracks might have been present during the earlier stage of the experiments. On the other hand, perhaps microcracks did not form in our experiments because the confining pressure was much lower than the normal stress applied in the Gratier et al. indenter study (~ 28 MPa in present test QC14 vs. up to 300 MPa in the Gratier et al. experiments).

### On the morphological differences of contacts between quartz grain and polished quartz crustal

**Figure 7:**
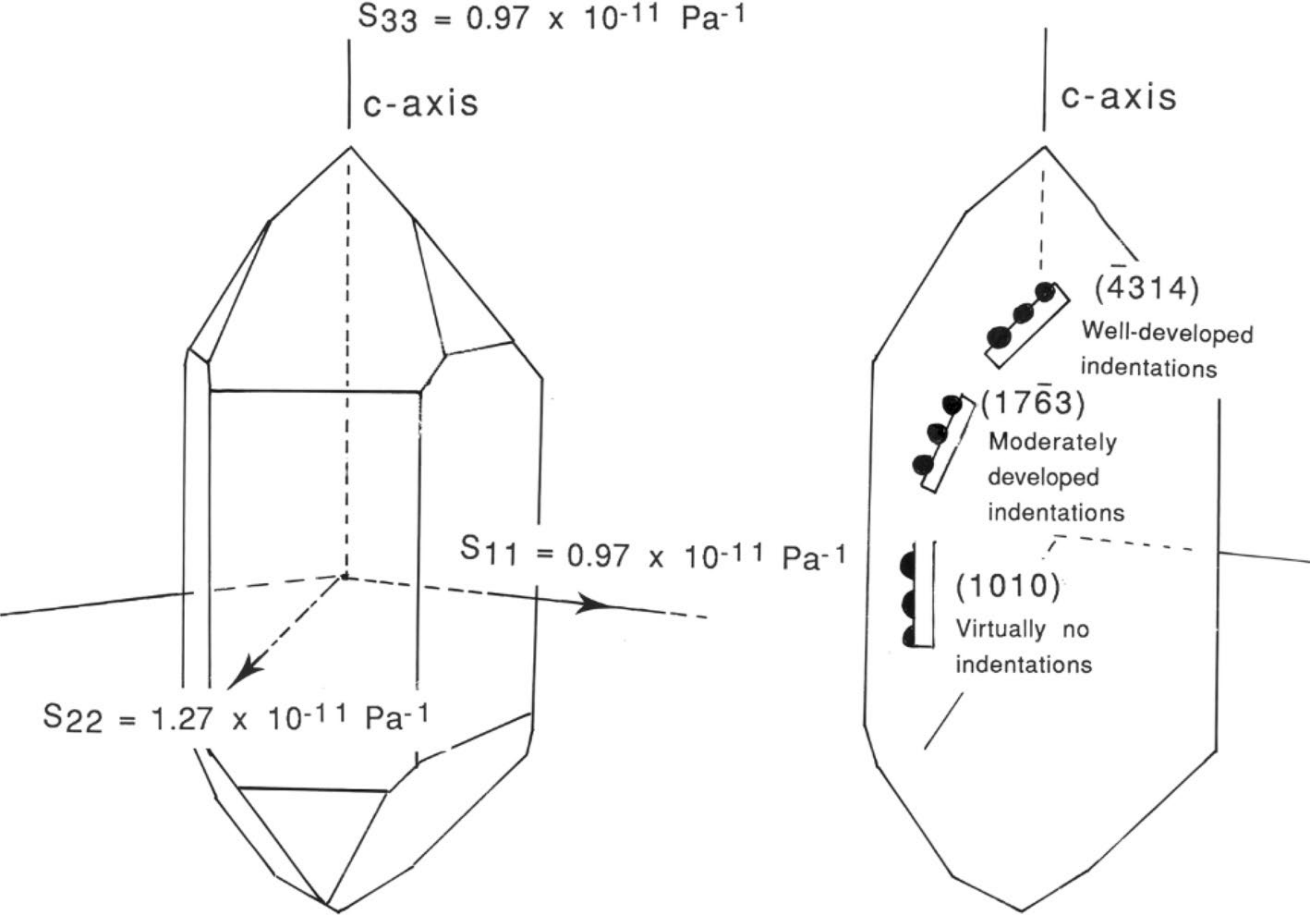
Schematic diagrams illustrating the variation in compliance in quartz with crystallographic orientation (**a**) and the degree of pit development of the quartz faces (**b**), oriented with respect to the c-axis of the Brazilian quartz crystal.

A substantial variation in indentation vs. truncation morphology was seen in tests QC13 and QC14. In test QC13 truncations were well developed, whereas in test QC14 indentation "pits" were apparently predominant (note that truncations at the indenting quartz grains cannot be ruled out). In addition, well-developed indentations developed in a quartz face ($\overline{4}314$) with normal at a small angle to the c-axis, whereas much smaller indentations were seen on the quartz face ($17\overline{6}3$) with normal at a higher angle to the c-axis (Fig. 7). If this apparent orientation-dependence of the supposed extent of IPS is a *real* orientation effect (as opposed to being related to the different shapes of the grains in the two sands used, or to differences in the chemical composition of the quartz sands, or to the hydrostatic versus 1D compaction set-ups used), it may be due to an influence of the crystal orientation on the kinetics of dissolution at the single crystal surface, or possibly even the driving force for pressure solution through a dependence of solid chemical potential on contact shear stress as well as normal stress^30,31^. The deduction of an effect of kinetics is supported by literature suggesting that the dissolution reaction kinetics of quartz in aqueous solutions may vary with crystallographic orientation (e.g.^32–34^. Kennedy^35^ found that quartz cut with poles parallel to the c-axis (i.e. basal planes) reach equilibrium with water a factor 2 to 4 faster than quartz plates with poles along or close to the c-axis (i.e. rhomb or prism faces). Hicks et al.^7^ concluded from a petrographic study on IPS in quartz-rich sandstone that, given a concave-convex contact between two quartz grains, the c-axis of the concave grain tends to lie at a lower angle to the pole of the contact plane than does that of the convex grain (Supplementary Fig. 5). Bons and Den Brok^27^ modeled the development of Crystal Preferred Orientations (CPOs) for the case of reaction-controlled pressure solution,—and only a twofold contrast in dissolution kinetics was needed to obtain CPOs similar to those observed in natural quartz-rich rocks^36,37^.

The above observations are in qualitative agreement with the present variation in inferred extent of IPS with crystallographic orientation of the polished quartz faces. However, it must be kept in mind that the variation in IPS microstructures may equally well (or partly) reflect the difference in experimental configurations, materials and testing conditions. More systematic experiments are needed to investigate the influence of experimental conditions on the IPS mechanism in the quartz-water system in greater detail and to assess the magnitude, systematics and mechanistic origin of orientation effects on IPS rates.

## Conclusions and recommendations

Polished quartz crystals embedded in wet quartz sand and axially or hydrostatically compacted in the presence of a fluid phase at a temperature of 350 °C and effective pressures of 15 MPa (axial loading) and 28 MPa (isotropic loading) show convincing evidence for IPS. This confirms predictions from mechanism-based models. Axial compaction gave truncation of quartz grains against the quartz plates. Hydrostatic compaction led to the development of indentations in the polished crystal faces and to the formation of overgrowths on the polished crystal faces. Truncations at indenting quartz grains may also have developed but to what extent is unknown. The morphology of the indentation pits and the truncations suggests the formation of these by grain boundary diffusional IPS rather than by marginal dissolution. The surface morphology of the indentations is characterized by protuberances, ridges and furrows with relief ranging from several hundred nanometers to a few microns. The surface morphology at the truncated contacts consists of inclusions of sub-rounded to crystallographically controlled shape separated by smooth intervening regions. The experimental observations agree with experimental and petrographic data suggesting crystallographic control of (stress-induced) dissolution rate, with relatively rapid dissolution kinetics at quartz face ($\overline{4}314$) and relatively slow dissolution kinetics at quartz face ($17\overline{6 }3$).

We report here on only two experiments, yet we hope that the microstructures and interpretation of the deformation mechanisms will stimulate further work. Our experiments are quite simple, i.e. they do not require a high-temperature triaxial deformation apparatus (a pressure cell that can be heated will do),—and the samples can be readily and cheaply made. This may facilitate further experimental research, e.g. with different hydrothermal quartz-water mixtures to apply different effective confining pressures on several gold-capsuled samples loaded in the same experiment. The crystallographic orientation of the polished quartz face in contact with the quartz grains in the matrix can then be varied in a systematic manner to study the effect of CPO on IPS. If such experiments reveal similar IPS-microstructures as the ones reported here, quantitative interpretation of the volume removed by indentation and volume deposited by precipitation should be attempted using e.g. image analysis techniques from digital-rock applications or 3D-imaging using multi-side illumination techniques. The impact of quartz-grain surface morphology on IPS can be investigated by using samples of different natural quartz sands with different grain surface rugosity than Bolderiaan or St. Peter. Advanced etching or grinding techniques can be applied to either flatten or enhance asperities on the quartz grain surface, to investigate how it affects the morphology of grain boundaries in truncation and indentation microstructures produced by IPS. New experiments should also investigate whether roughness/furrows in the floor /faces of the pits/contacts (as in experiment QC14) are associated with cracks penetrating into the quartz crystal, ref. work by Renton et al.^19^ and Gratier et al.^16^.

In order to extrapolate experimental results to natural conditions, the full formulation of the serial processes of dissolution, diffusion and precipitation must be used to account for possible fluctuations in the rates of either of these processes. Clearly, to improve the understanding of the kinetics of the mechanisms operating during IPS in natural wet quartz-rich rocks, more detailed experiments are needed. Further insight in the potential importance of variability in dissolution, precipitation and diffusion rates for different crystal faces can be obtained from experimental and microstructural data combined with mechanism-based models in which the thermodynamics and kinetics of the pressure-solution process in water-saturated quartz sands is taken into account.

## Supplementary Information

Supplementary Information.
