# Supplementary material for: Surface microstructures developed on polished quartz crystals embedded in wet quartz sand compacted under hydrothermal conditions
Source: Sci Rep. 2021 Jul 21;11:14920. doi: 10.1038/s41598-021-94376-1 (PMC8295331; doi:10.1038/s41598-021-94376-1)
Supplement: Supplementary file 1 — Supplementary Information. [file 41598_2021_94376_MOESM1_ESM.docx]

Supplementary material to
“Surface microstructures developed on polished quartz crystals embedded in wet quartz sand compacted under hydrothermal conditions”

by Peter M. Schutjens, Christopher J. Spiers and André R. Niemeijer

Nature Scientific Reports

This material contains five additional figures:

*Supplementary figure 1*

*Diagrams illustrating the mechanisms of grain indentation by intergranular pressure solution. The evolution of grain contact microstructure with time is represented by four drawings (left to right).
Top: marginal dissolution. with the hatched area representing the region of plastic deformation or micro-granulation.
Middle and bottom: grain boundary diffusional intergranular pressure solution. with transport through a thin adsorbed film or island-channel structure (indicated), after [1]Raj (1982).*

*Supplementary figure 2.*

*Schematic illustration showing direction at which the quartz cylinders (diameter= 1*

*cm) were cored from the Brazilian quartz crystal and the way the cylinder was*

*sectioned to smaller parts. Crystallographic directions r, m and c are indicated.*

*Supplementary figure 3.*

*(a) SEM micrograph of polished quartz crystal face (10*$\bar{1}$*0) before hydrostatic compaction experiment.*

*(b) Characteristic SEM micrograph of "Bolderiaan" quartz sand after the acid treatment and washings with NaOH-solutions and distilled water.*

*(c) Diagram showing the configuration of the polished quartz single crystals in compaction experiments QC13. The quartz sand surrounding the polished crystals is not indicated, but in practice fills up the remaining space in the sample chamber.*

*Supplementary figure 4*

*(a, b) SEM micrographs of characteristic grains from the St. Peter sandstone formation. Micrograph 4a was taken before ultrasonic cleaning of the quartz grain surface. Micrograph 4b was taken after the ultrasonic cleaning.*

*(c) Diagram showing the configuration of the polished quartz single crystal in compaction experiments QC14. A quarter cylinder with two polished faces (*$\bar{4}$*314) and (17*$\bar{6}$*3) is embedded in quartz sand (not indicated, but in practice filling up the remaining space in the capsule).*

*Supplementary figure 5.*

*The observations of Hicks et at [2] suggesting crystallographic control on IPS in natural rocks. The c-axis of concave quartz grains tends to lie at a lower angle to the pole of the contract plane than does that of the contacting convex grain. This observation agrees with the present experimental observations.*

References cited in Supplement:

[1] Raj. (1982). Creep in polycrystalline aggregates by matter transport through a liquid phase. Journal of Geophysical Research: Solid Earth, 87(B6), 4731-4739. doi:10.1029/JB087iB06p04731

[2] Hicks, Applin, K. R., & Houseknecht, D. W. (1986). Crystallographic influences on intergranular pressure solution in a quartzose sandstone. Journal of Sedimentary Research, 56(6), 784. doi:10.1306/212F8A49-2B24-11D7-8648000102C1865D





*Supplementary figure 1*


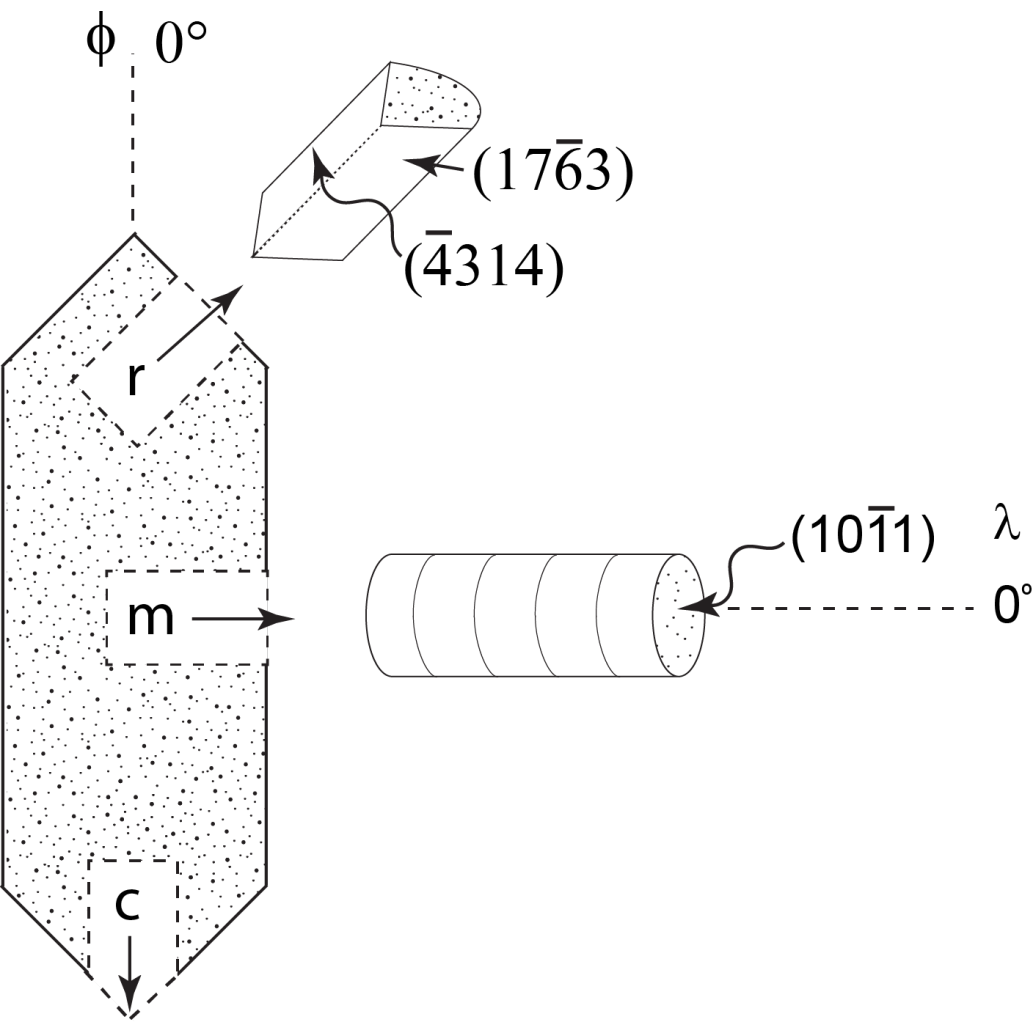


*Supplementary figure 2*


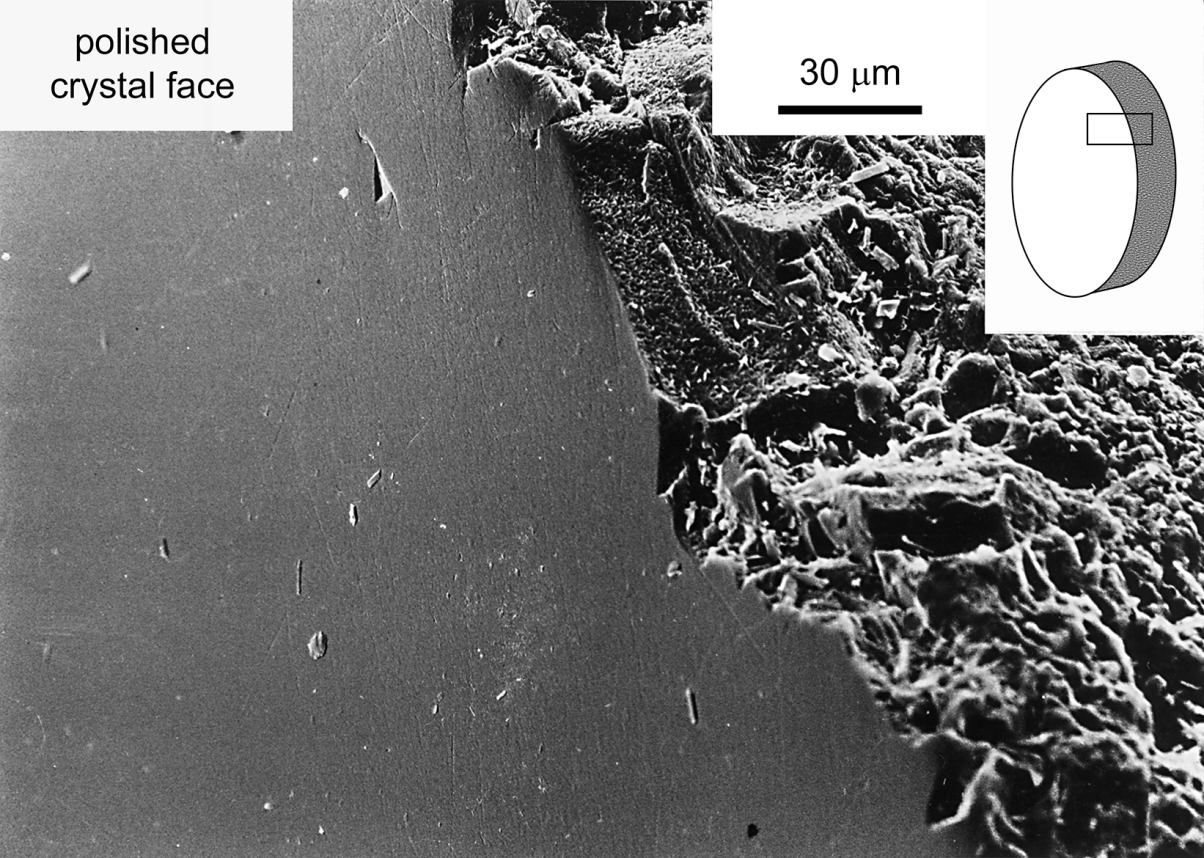


*Supplementary figure 3a*


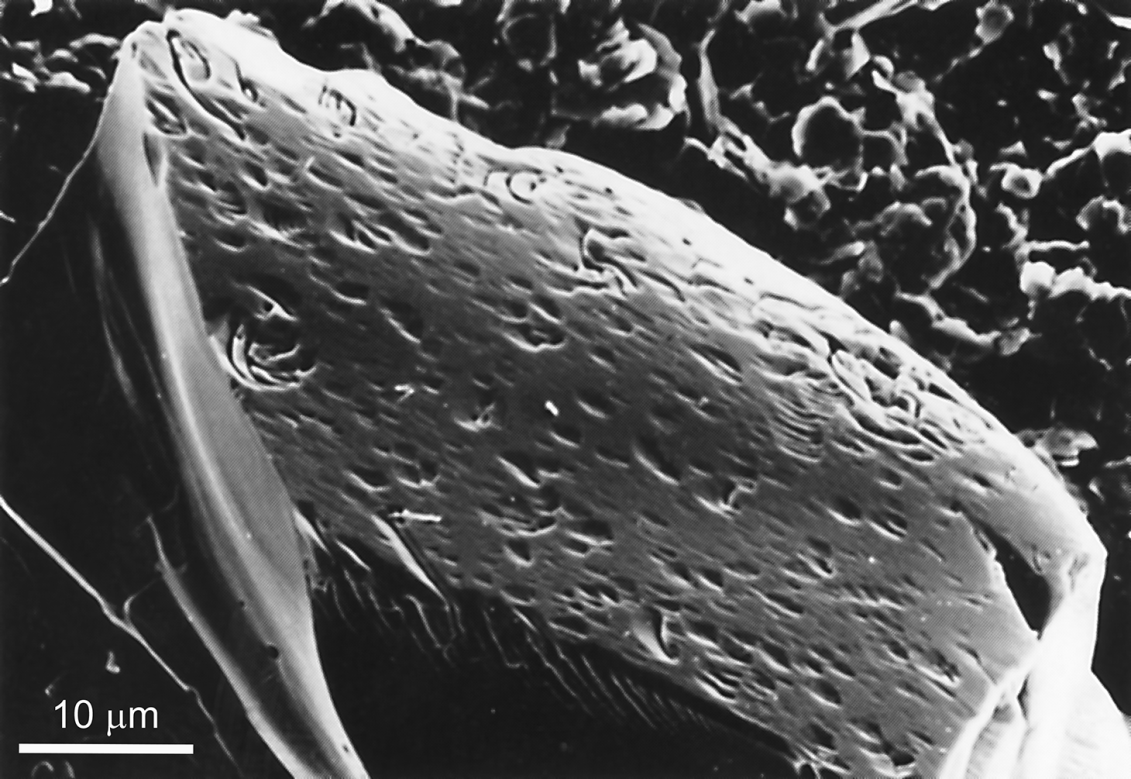


*Supplementary figure 3b*


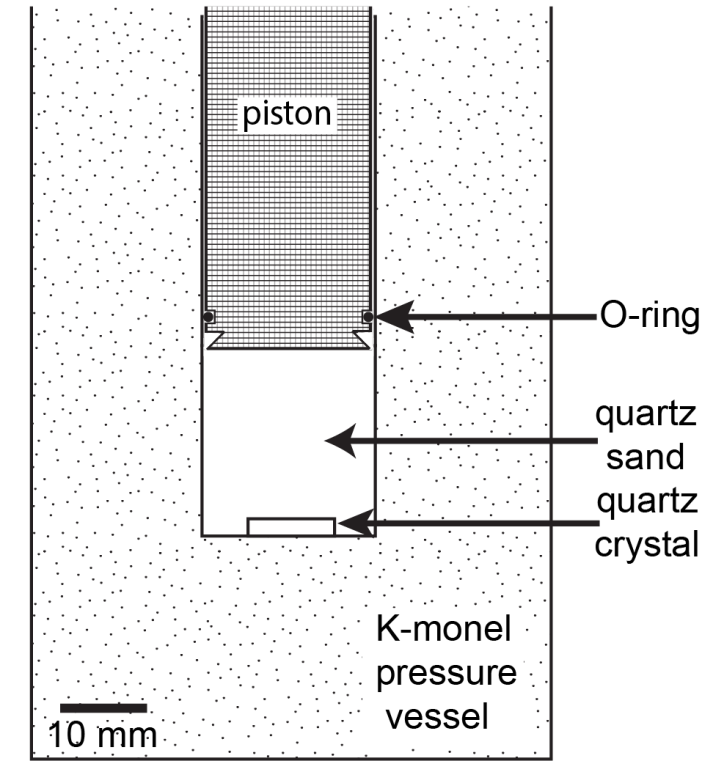


*Supplementary figure 3c*


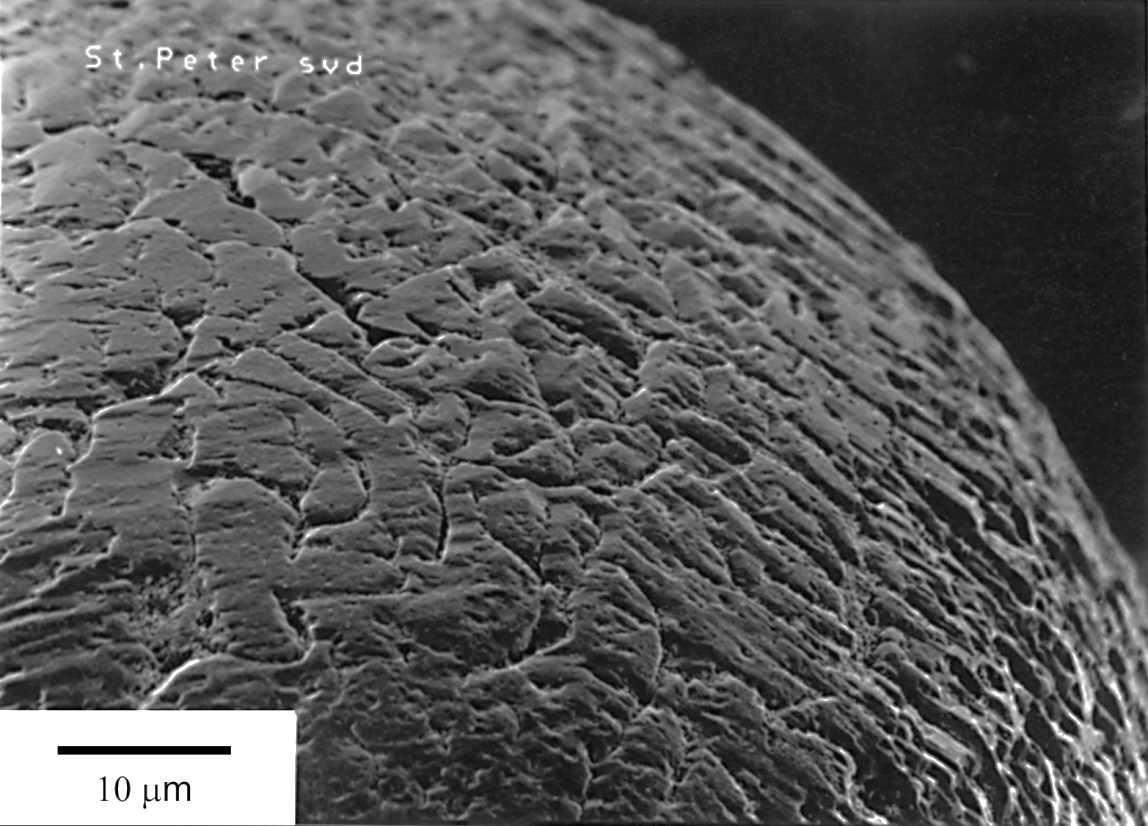


*Supplementary figure 4a*


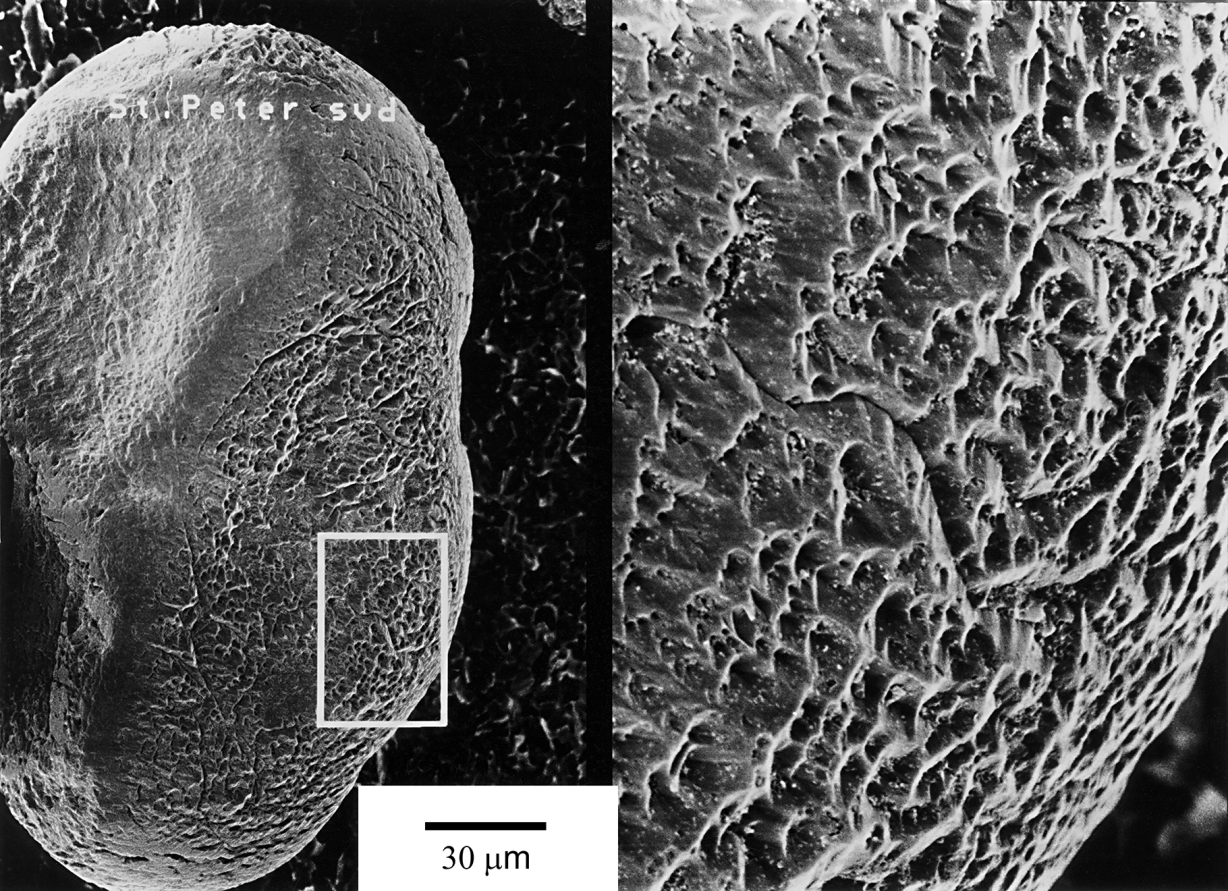


*Supplementary figure 4b*


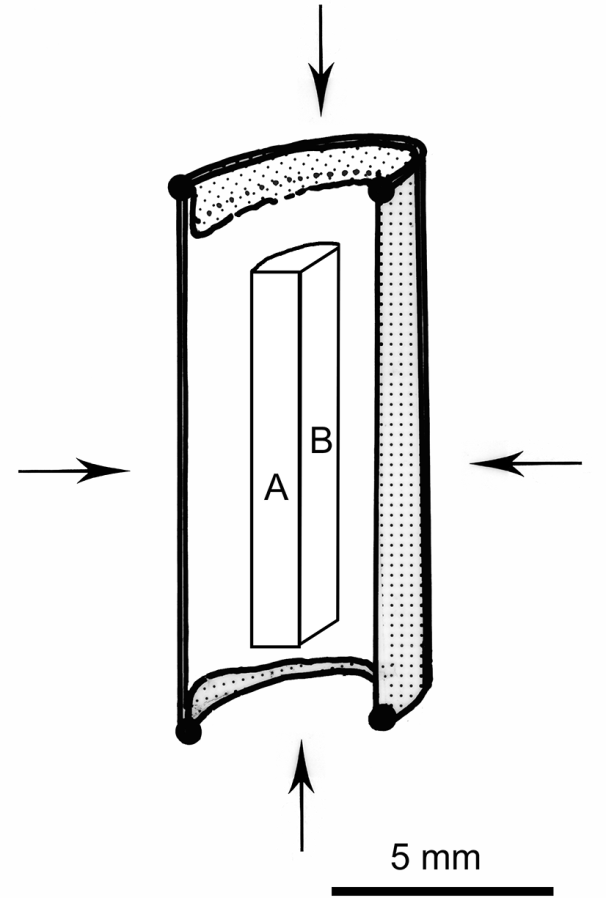


*Supplementary figure 4c*

*
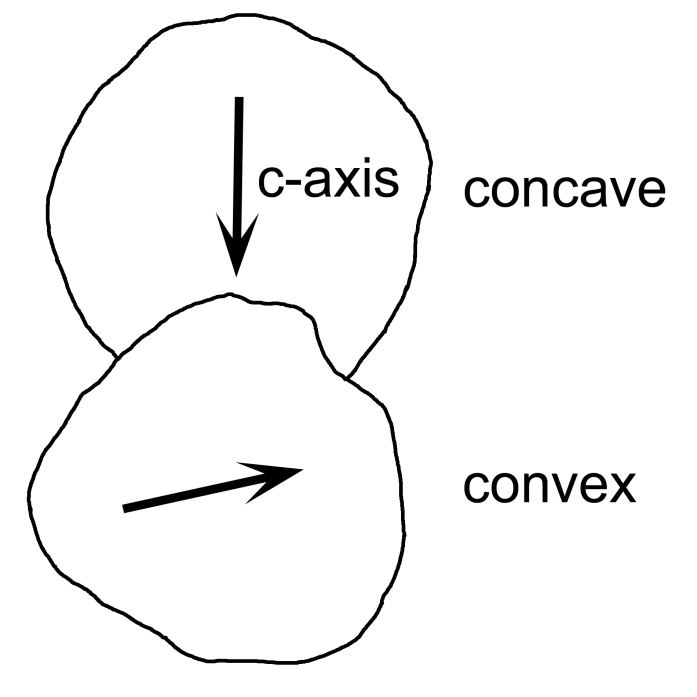
*

*Supplementary figure 5*
